# Prognostic significance of myocardial salvage assessed by cardiac magnetic resonance in reperfused ST-segment elevation myocardial infarction

**DOI:** 10.3389/fcvm.2022.924428

**Published:** 2022-08-30

**Authors:** Yunling Li, Guokun Wang, Xueying Wang, Ye Li, Yanming Zhao, Xia Gu, Bing Xu, Jinjin Cui, Xuedong Wang, Yong Sun, Shengliang Liu, Bo Yu

**Affiliations:** ^1^Department of Cardiology, Cardiovascular Imaging Center, The Second Affiliated Hospital of Harbin Medical University, Harbin, China; ^2^The Key Laboratory of Myocardial Ischemia, Chinese Ministry of Education, Department of Cardiology, The Second Affiliated Hospital of Harbin Medical University, Harbin, China

**Keywords:** acute myocardial infarction, cardiac magnetic resonance, myocardial salvage index, infarct size, area at risk

## Abstract

**Aims:**

Myocardial salvage index (MSI) is attracting increasing attention for predicting prognosis in acute myocardial infarction (AMI); however, the evaluation of MSI is mainly based on contrast agent-dependent cardiac magnetic resonance (CMR) scanning sequences. This study aims to investigate the prognostic value of MSI in reperfused ST-segment elevation myocardial infarction (STEMI) through the contrast agent-free CMR technique.

**Methods and results:**

Nighty-two patients with acute STEMI, who underwent CMR after primary percutaneous coronary intervention (PPCI), were finally enrolled. Patients were subcategorized into two groups according to median MSI. T1 and T2 mapping were conducted for measuring infarct size (IS) and area at risk (AAR). IS was significantly larger in < median MSI group than ≥ median MSI group (*P* < 0.001). AAR between the two groups showed no obvious differences (*P* = 0.108). Left ventricular ejection fraction (LVEF) was lower in < median MSI group than ≥ median MSI group (*P* = 0.014). There was an obvious inverse correlation between MSI and reperfusion time (*R* = –0.440, *P* < 0.001) and a strong inverse correlation between MSI and IS (*R* = –0.716, *P* = 0.011). As for the relationship LVEF, MSI showed positive but weak correlation (*R* = 0.2265, *P* < 0.001). Over a median follow-up period of 263 (227–238) days, prevalence of MACEs was significantly higher in the < median MSI group [HR: 0.15 (0.04–0.62); Log-rank *P* = 0.008]. The univariate Cox regression analysis revealed that LVEF, IS, and MSI were significant predictors for major adverse cardiovascular events (MACEs) (all *P* < 0.05). In the stepwise multivariate Cox regression analysis, LVEF and MSI were identified as independent parameters for predicting MACEs (both *P* < 0.05). In the receiver-operating characteristic analysis, LVEF, IS, and MSI showed prognostic value in predicting MACEs with AUCs of 0.809, 0.779, and 0.896, respectively, all (*P* < 0.05). A combination of MSI with LVEF showed the strongest prognostic value of MACEs (AUC: 0.901, sensitivity: 77.78%, specificity: 98.80%, *P* < 0.001). Delong’s test showed that the combination of LVEF with MSI had an incremental value than LVEF itself in predicting MACEs (*P* = 0.026).

**Conclusion:**

Contrast agent-free CMR technique provides a reliable evaluation of MSI, which contributes to assessing the efficacy of reperfusion therapy and predicting the occurrence of MACEs.

## Introduction

Myocardial salvage index (MSI) is attracting more and more attention for its superiority in assessing the efficacy of reperfusion therapy in acute myocardial infarction (AMI) (1). Reperfusion following AMI is mainly constituted by pharmacological thrombolysis, primary percutaneous coronary intervention (PPCI), and coronary artery bypass grafting, all of which are responsible for effective myocardial salvage. Infarct-related parameters are associated with adverse clinical outcomes; quantification of myocardial injury is necessary for evaluating treatment effects (2). The amount of salvaged myocardium is closely related to the initial myocardial area at risk (AAR) and irreversible infarct myocardium (3), difference between edema-based AAR and infarct area is used to calculate MSI (4). Absolute myocardial infarct size (IS) has been utilized to predict the prognosis of AMI; however, many patients develop extensive myocardial damage even after receiving revascularization by PPCI (5), which draws attention to reversible myocardial injury; thus, MSI might be an effective indicator in predicting AMI prognosis (6).

Single-photon emission computed tomography (SPECT) has been widely confirmed in detecting MSI (7–9), which is an independent predictor of prognosis in AMI (10). However, this technique is unreasonable in an acute condition, as it requires injection of isotope before reperfusion. In addition, inevitable radiation exposure and low spatial resolution make it limited in clinical practice (9). Thus, developing novel and feasible methods to measure MSI and assess reperfusion efficiency is of utmost importance.

Cardiac magnetic resonance (CMR) is a radiation-free and multi-parametric imaging technique with high sensitivity and resolution. It offers reproducible measurement of MSI with excellent consistency with SPECT (7, 11). A previous study proved that combining T2-weighted imaging and late gadolinium enhancement (LGE) offered stable MSI measurement (4). However, contrast agent-mediated LGE imaging is limited to a certain extent, as many patients with AMI also carry chronic kidney diseases simultaneously, in addition, contrast-induced nephropathy following PPCI cannot be ignored as well. Besides that, a comprehensive CMR can be challenging and time-consuming for patients with AMI, obviating some scanning protocols and shortening scanning duration without compromising data acquisition would be an ideal approach (12). Native T1 mapping can identify myocardial fibrosis and quantify IS without utilizing gadolinium agents (13–15), which gives an estimate of IS for particular patient groups. A high T2 signal reflects an increased myocardial water content; T2 mapping is confirmed effective in assessing myocardial edema, which is considered as AAR in AMI (16). Comparing and quantifying T1 and T2 mapping ideally provide MSI and assess reperfusion efficacy.

The purpose of the study is to investigate the prognostic value of MSI in reperfused (AMI) through the contrast agent-free CMR technique.

## Materials and methods

### Study population

This retrospective trial was performed at a single cardiac center between September 2020 and November 2021. Patients were eligible if symptoms lasted less than 12 h, and the ST-segment elevated more than 0.1 mV in at least two extremity leads or more than 0.2 mV in at least two precordial leads (17). Patients with first ST-segment elevation myocardial infarction (STEMI) undergoing PPCI were initially enrolled. The CMR study was carried out within 5 days of PPCI. Patients with previous myocardial infarction or contraindications to CMR, such as implanted pacemakers, defibrillators, claustrophobia, or metallic intracranial implants, were excluded. The study was approved by the local ethics committee and all patients gave written informed consent.

### Primary percutaneous coronary intervention and subsequent treatment

Prior to PPCI, all patients orally received a 300-mg loading dose of aspirin and clopidogrel separately. In addition, they received low molecular heparin xintravenously. All patients received PPCI according to standard clinical practice, additional use of thrombectomy was performed depending on the thrombus burden in an occluded artery. After PPCI, aspirin and clopidogrel were administered at a dose of 100 and 75 mg, respectively, per day. All other medications, including glycoprotein IIb/IIIa-inhibitors, angiotensin-converting enzyme inhibitors, beta-blockers, and statins, were recommended according to contemporary guidelines (18).

### Cardiac magnetic resonance acquisition

CMR imaging was performed in all patients by using a 3.0-Tesla system (Ingenia CX, Philips Healthcare, Netherlands), with a 32-channel phased-array abdomen coil. The scanning protocols mainly included cine steady-state free precession imaging, T2-weighted short tau inversion recovery (T2w-STIR), native T1 mapping, T2 mapping, and LGE.

After performing scout imaging, cine imaging with breath-hold and electrocardiogram trigger were used for cardiac morphologic and functional analyses. The scanning was conducted in short-axis slices, 2-chamber, 3-chamber, and 4-chamber planes, for short-axis imaging; left ventricular (LV) was completely encompassed from base to apex, and the parameters were as follows: repetition time (TR)/echo time (TE) = 2.8/1.42 ms, the field of view (FOV) = 300 × 300 mm^2^, voxel = 1.8 × 1.6 × 8 mm^3^, flip angle = 45°, and 8-mm slice thickness.

For T2w-STIR imaging, a T2-weighted imaging triple inversion recovery turbo spin-echo sequence based on breath hold was applied; the scanning parameters were as follows: TR/TE = 1,500/75 ms, FOV = 300 × 300 mm^2^, voxel = 1.3 × 1.65 × 8 mm^3^, and flip angel = 90°. The T2w-STIR images were acquired on short-axis planes including the base, mid-ventricular, and apex levels.

Modified Look-Locker inversion recovery sequence was performed for native T1 mapping in short-axis slices (basal, mid, and apical) (15, 19); the detailed scanning parameters were as follows: TR/TE = 2.2/1.02 ms, FOV = 300 × 300 mm^2^, voxel = 2 × 2 × 8 mm^3^, flip angle = 20°, and 8-mm slice thickness.

T2 mapping was acquired in a gradient-spin echo sequence (20, 21) at the same short-axis positions corresponding to T1 mapping, which included basal, mid, and apical LV. The sequence parameters were TR/TE = 741/8.8 ms, FOV = 300 × 300 mm^2^, voxel = 2 × 2 × 8 mm^3^, flip angel = 90°, and 8-mm slice thickness.

LGE images were acquired 10–15 min after injection of gadolinium-based contrast agent (Bayer Healthcare, Germany) by using a three-dimensional phase-sensitive inversion recovery sequence (22), the scanning parameters of LGE were TR/TE = 6.1/3 ms, FOV = 300 × 300 mm^2^, voxel = 1.8 × 1.68 × 8 mm^3^, flip angel = 25°, and 8-mm slice thickness. The inversion time was optimized to null the signal in the normal myocardium.

### Cardiac magnetic resonance analysis

**FIGURE 1:**
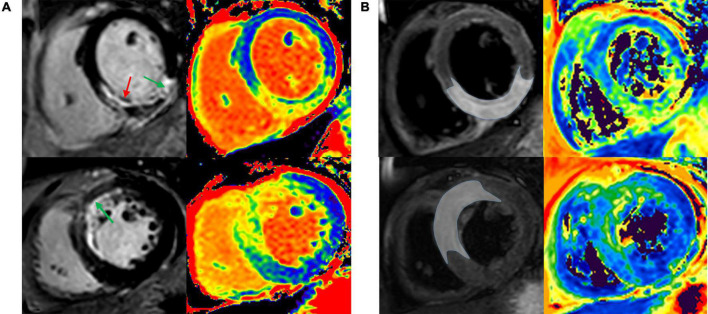
Infarct region and AAR were validated by comparing T1 and T2 mapping against LGE and T2w-STIR. Representative images of patient with acute anterior and anteroseptal STEMI **(A)**, and patient with acute inferior and inferoseptal STEMI **(B)**. AAR, area at risk; LGE, late gadolinium enhancement; T2w-STIR, T2-weighted short tau inversion recovery; STEMI, ST-segment elevation myocardial infarction.

All CMR studies were post-processed with a dedicated workstation (cvi42, Circle Cardiovascular Imaging Inc., Calgary, Alberta, Canada) by two experienced operators who were blinded to baseline and outcome data. LV dimensions, mass, and systolic function were calculated from the SSFP cine. Typical CMR images of patients with acute anterior, anteroseptal myocardial infarction and inferior, and inferoseptal myocardial infarction are shown in Figure 1. Validation of infarct region and edema-based AAR are obtained by comparing T1 mapping, T2 mapping against LGE, and T2w-STIR as the reference standards.

### Measurement of infarct area in T1 mapping

For native T1 mapping, pixel-coded T1 values were provided in a parametric color-encoded anatomical map, followed by analyses that were consistent with current guidelines (23). Epicardial and endocardial contours of LV and the remote myocardium without visible evidence of infarction, edema, or abnormal motion (assessed through LGE, T2w-STIR, and cine images) were delineated. Regions of interest, which are the infarct areas, were defined as myocardium with a signal-intensity threshold of > 5 standard deviations (SDs) above remote myocardium (24). The hypo-intense infarct cores that represented microvascular damage were also included within IS after manual delineation.

### Measurement of the area at risk in T2 mapping

T2 mapping sequences provide T2 value per pixel in milliseconds and deliver a good correlation with myocardial water content. AAR was determined when pixel values of myocardium > 2 SDs above remote myocardium (25, 26). In T2 mapping, the epi- and endocardial contours and remote myocardium were correspondingly copied from T1 mapping. Central hypo-intense cores that were deemed to be myocardial hemorrhage were included in the AAR measurement (27). Attention should be paid to excluding high signals caused by slow flow in the blood pool. IS and AAR were normalized as a percentage of LV mass. The following formulas were applied: AAR = volume edema/volume LV mass, IS = volume infarct/volume LV mass, MSI = (AAR–IS)/AAR (4, 24).

### Clinical endpoints and definitions

The primary endpoint of this study was defined as the emergence of major adverse cardiovascular events (MACEs), which included all-cause death, re-infarction, and new congestive heart failure (1). Re-infarction was diagnosed based on ischemic symptoms, new ST-segment changes, increase in creatine kinase, and troponin I. New congestive heart failure was defined according to New York Heart Association functional class. Clinical follow-up was conducted *via* a structured questionnaire by telephone and then assessed by two experienced cardiologists. The telephone interview was conducted every 6 months after the CMR test.

### Statistical analysis

Categorical variables were expressed as numbers, with percentages in parentheses, and differences were assessed by the Fisher exact or Chi-square test. Continuous data with normal distribution were compared through Student’s *t*-test and expressed as mean ± SD, continuous variables with non-normal distribution were compared by the Mann-Whitney *U*-test and were, therefore, presented as medians with the interquartile range. Pearson’s or Spearman’s correlation coefficients were calculated to assess the correlations between left ventricular ejection fraction (LVEF) and infarct-related parameters. Univariate and multivariate COX regression analyses were performed to calculate hazard ratio (HR) and 95% CI, then characterize predictors of MSI. The Kaplan-Meier method was used for depicting survival curves; differences were assessed by log-rank tests. The area under the curves (AUCs), specificity, sensitivity, and Youden’s index were analyzed by the receiver-operating characteristic curve to define optimal cut-off values for the prediction of clinical endpoints. Intra- and interobserver variabilities were assessed using intraclass correlation coefficients (ICC). Data above were calculated by SPSS 26.0.0 (Inc., Chicago, IL, United States) or by the MedCalc version20 (MedCalc Software, Ostend, Belgium). *P* < 0.05 was statistically significant.

## Results

### Study population

**FIGURE 2:**
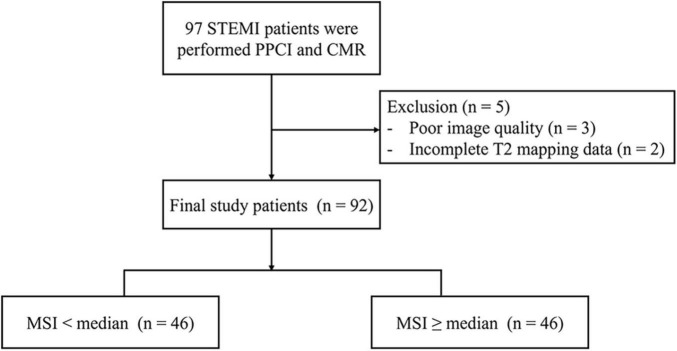
Study flow diagram.

This retrospective study consisted of 97 consecutive patients with STEMI, 3 patients were excluded because of poor image quality, and 2 patients were excluded because of lacking T2 mapping (Figure 2). Clinical data of the rest of the 92 patients were collected, and images exhibited diagnostic quality, enabling the assessment of myocardial salvage. PPCI was performed in the left anterior descending artery in 47 patients, in the left circumflex artery in 42 patients, and in the right coronary artery in 3 patients. Demographic data and clinical characteristics are presented in Table 1. Patients were separated into two groups according to median MSI, that was a < median MSI group (*n* = 46) and a ≥ median MSI group (*n* = 46). The baseline characteristics (age, sex, body mass index) and risk factors (hypertension, diabetes mellitus, and smoking) were similar between the two groups (all *P* > 0.05). The mean age of the patients with STEMI was 59 ± 10 years old and 75% were male. The ≥ median MSI group tended to show lower level of cholesterol and shorter time from symptom onset to reperfusion in comparison to < median MSI group [cholesterol, 4.6 ± 1 mmol/L vs. 5.1 ± 1.1 mmol/L; reperfusion time, 180 (120, 360) min vs. 330 (180, 480) min; both *P* < 0.05]. Levels of N-terminal pro-B type natriuretic peptide (NT-proBNP) and peak troponin I of the two groups showed no significant differences [NT-proBNP, 334.5 (97.5, 823.8) pg/ml vs. 405 (100.8, 1,422.5) pg/mL; troponin I, 18.2 (6.7, 85.5) ng/ml vs. 44.9 (11.7, 117.2) ng/mL; both *P* > 0.05]. There were no statistical differences between the two groups in terms of involved culprit coronary arteries (*P* = 0.641). Concomitant medications after PPCI between the two groups were similar except for nitrates.

**TABLE 1:** Demographic data and clinical characteristics of patients with STEMI.

Characteristics	All patients (*n* = 92)	<Median MSI (*n* = 46)	≥Median MSI (*n* = 46)	*p*-value
Age (years)	59 ± 10	58 ± 11	59 ± 10	0.735
Male gender, *n* (%)	69 (75.0)	37 (80.4)	32 (69.6)	0.229
BMI (kg/m^2^)	25.4 ± 3.9	25.8 ± 4.2	25.0 ± 3.7	0.354
Symptom onset to reperfusion (min)	240 (180–420)	330 (180–480)	180 (120–360)	0.001
PPCI to CMR (days)	3.9 (3.0–4.8)	3.6 (3.0–4.7)	4.0 (3.0–4.9)	0.316
**Medical history**				
Hypertension, *n* (%)	31 (33.7)	16 (34.8)	15 (32.6)	0.825
Smoking, *n* (%)	41 (44.6)	21 (45.7)	20 (43.5)	0.834
Diabetes mellitus, *n* (%)	13 (14.1)	5 (10.9)	6 (17.4)	0.369
**Culprit coronary artery**				0.641
LAD, *n* (%)	47 (51.1)	22 (47.8)	25 (54.3)	
RCA, *n* (%)	3 (3.3)	1 (2.2)	2 (4.3)	
LCX, *n* (%)	42 (45.7)	23 (50.0)	19 (41.3)	
**Initial blood results on admission**				
NT-proBNP (pg/mL)	351.5 (101.0–1110.3)	334.5 (97.5–823.8)	405.0 (100.8–1422.5)	0.380
Peak troponin I, (ng/mL)	29.7 (8.9–107.0)	18.2 (6.7–85.5)	44.9 (11.7–117.2)	0.120
Cholesterol	4.8 ± 1.1	5.1 ± 1.1	4.6 ± 1.0	0.021
**Medications post-PPCI**				
ACE-I or ARB, *n* (%)	50 (54.3)	26 (56.5)	24 (52.2)	0.675
Beta blocker, *n* (%)	63 (68.5)	29 (63.0)	34 (73.9)	0.262
Statins, *n* (%)	90 (97.8)	45 (97.8)	45 (97.8)	1.000
Aspirin, *n* (%)	92 (100.0)	46 (100.0)	46 (100.0)	1.000
Nitrates, *n* (%)	30 (32.6)	10 (21.7)	20 (43.5)	0.026
Clopidogrel, *n* (%)	87 (94.6)	44 (95.7)	43 (93.5)	1.000
Ticagrelor, *n* (%)	3 (3.3)	1 (2.2)	2 (4.3)	1.000

*P* < 0.05 is considered to indicate statistical significance. STEMI, ST-segment elevation myocardial infarction; MSI, myocardial salvage index; BMI, body mass index; LAD, left anterior descending; RCA, right coronary artery; LCX, left circumflex artery, NT-proBNP, N-terminal pro-B type natriuretic peptide; ACEI, angiotensin-converting enzyme inhibitor; ARB, angiotensin receptor blocker.

### Cardiac magnetic resonance

The median time between PPCI and CMR acquisition was 3.9 (3–4.8) days for the whole study group, 3.6 (3–4.7) days for < median MSI group, and 4 (3–4.9) days for ≥ median MSI group. CMR parameters are summarized in Table 2. Regions of increased signal intensity on native T1 and T2 mapping were measured in the territory of corresponding occluded coronary arteries. Mean IS among all the patients was 22.6 ± 12% of LV mass. IS was significantly larger in the < median MSI group relative to ≥ median MSI group (29.2 ± 11.6% vs. 15.9 ± 8.2%, *P* < 0.001). LGE was more prevalent in the < median MSI group than ≥ median MSI group (28.1 ± 13.5% vs. 16.9 ± 8%, *P* < 0.001). The mean edema-based AAR of the whole studying cohort was 40.7 ± 14.7% of LV mass. AAR between the two groups showed no obvious differences (38.2 ± 13.7% vs. 43.2 ± 15.3%, *P* = 0.108). LV mass, end-diastolic volume (EDV), and end-systolic volume (ESV) were similar in the two groups (LV mass, 113.5 ± 26.7 g vs. 107.9 ± 24.9 g; LV EDV, 154.7 ± 35.8 ml vs. 148.2 ± 31.4 ml; LV ESV, 77.8 ± 27.6 ml vs. 73 ± 27.8 ml; all *P* > 0.05); whereas LVEF was obviously lower in the < median MSI group than that in the ≥ median MSI group (51.5 ± 6.7% vs. 54.5 ± 5%, *P* = 0.014).

**TABLE 2:** CMR characteristics of patients with STEMI.

	All patients (*n* = 92)	<Median MSI (*n* = 46)	≥Median MSI (*n* = 46)	*p*-value
Heart rate (bmp)	71 ± 12	70 ± 11	71 ± 12	0.665
LV EDV, (ml)	151.5 ± 33.6	154.7 ± 35.8	148.2 ± 31.4	0.351
LV ESV, (ml)	75.4 ± 27.7	77.8 ± 27.6	73.0 ± 27.8	0.401
EF, (%)	53.0 ± 6.1	51.5 ± 6.7	54.5 ± 5.0	0.014
LV mass, (g)	110.7 ± 25.8	113.5 ± 26.7	107.9 ± 24.9	0.296
Infarct size, (% LV)	22.6 ± 12	29.2 ± 11.6	15.9 ± 8.2	<0.001
Area at risk, (% LV)	40.7 ± 14.7	38.2 ± 13.7	43.2 ± 15.3	0.108
LGE, (% LV)	22.5 ± 12.4	28.1 ± 13.5	16.9 ± 8.0	<0.001

*P* < 0.05 is considered to indicate statistical significance. CMR, cardiac magnetic resonance; STEMI, ST-segment elevation myocardial infarction; MSI, myocardial salvage index; LV, left ventricular; EDV, end-diastolic volume; ESV, end-systolic volume; EF, ejection fraction; LGE, late gadolinium enhancement.

### Correlations

**FIGURE 3:**
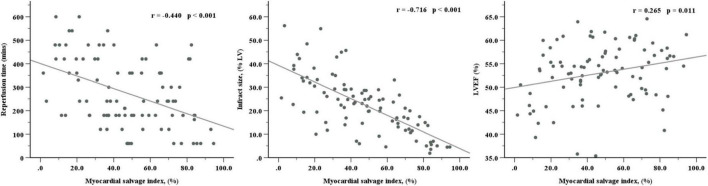
Correlations of myocardial salvage index with reperfusion time, infarct size, and LVEF. LVEF, left ventricular ejection fraction.

There was an obvious inverse correlation between MSI and reperfusion time (*R* = –0.440, *P* < 0.001) and a strong inverse correlation between MSI and IS (*R* = –0.716, *P* < 0.001). As for the relationship with LVEF, MSI showed a positive but weak correlation (*R* = 0.265, *P* = 0.011) (Figure 3).

### Clinical outcome

**FIGURE 4:**
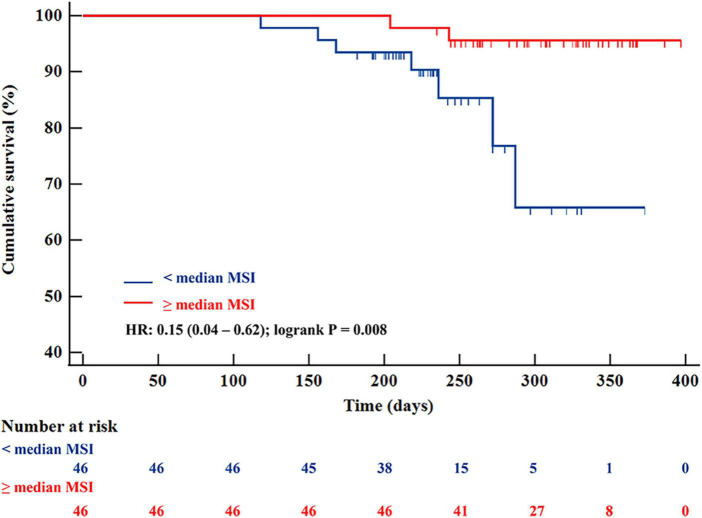
Kaplan-Meier survival curves for 92 patients with STEMI separated into two groups according to median MSI. STEMI, ST-segment elevation myocardial infarction; MSI, myocardial salvage index.

**FIGURE 5:**
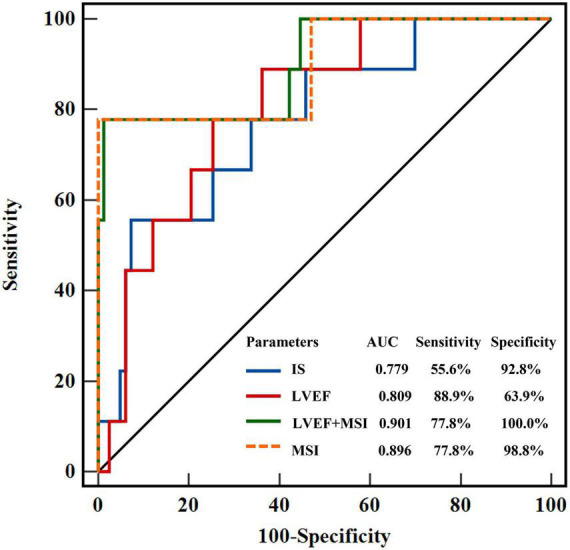
Receiver-operating characteristic curves showed the prognostic value of LVEF, MSI, IS, and the combined model of LVEF + MSI. LVEF, left ventricular ejection fraction; MSI, myocardial salvage index, IS, infarct size.

Over a median duration follow-up of 263 (227–238) days, the primary endpoint occurred in 9 patients; we observed 9 re-infarctions, among which there were 7 re-infarctions (15.2%) in the < median MSI group and 2 (4.3%) in the ≥ median MSI group. Cardiac death and congestive heart failure were not detected in both groups. Prevalence of MACEs was significantly higher in the < median MSI group (HR: 0.15 [0.04–0.62]; Log-rank *P* = 0.008) (Figure 4). Several CMR parameters (LVEF, IS, and MSI) were associated with MACEs by univariate COX regression analysis (all *P* < 0.05), while MSI and LVEF were the independent parameters in predicting clinical endpoint in further multivariate COX regression analysis (both *P* < 0.05) (Table 3). In the receiver-operating characteristic analysis, LVEF, IS, and MSI showed prognostic value in predicting MACEs with AUCs of 0.809, 0.779, and 0.896, respectively, (all *P* < 0.05). A combination of MSI with LVEF showed the strongest prognostic value of MACEs (AUC: 0.901, sensitivity: 77.78%, specificity: 98.80%, *P* < 0.001) (Figure 5). Delong’s test showed that the combination of LVEF with MSI had an incremental value than LVEF itself in predicting MACEs (*P* = 0.026).

**TABLE 3:** Univariate and stepwise multivariate cox regression analysis for the prediction of MACEs.

Variate	Univariate		Multivariate	
	Hazard ratio (CI)	*p*-value	Hazard ratio (CI)	*p*-value
EF, (%)	0.862 (0.789–0.943)	0.001	0.874 (0.775–0.985)	0.027
Infarct size, (% LV)	1.093 (1.038–1.150)	0.001	1.031 (0.961–1.106)	0.393
Area at risk, (% LV)	1.007 (0.961–1.056)	0.767	–	–
Reperfusion time, (min)	1.003 (0.999–1.007)	0.169	–	–
MSI, (%)	0.908 (0.861–0.958)	0.000	0.926 (0.875–0.980)	0.008

*P* < 0.05 is considered to indicate statistical significance. MACE, major adverse cardiovascular events; EF, ejection fraction; MSI, myocardial salvage index.

### Reproducibility analysis

**FIGURE 6:**
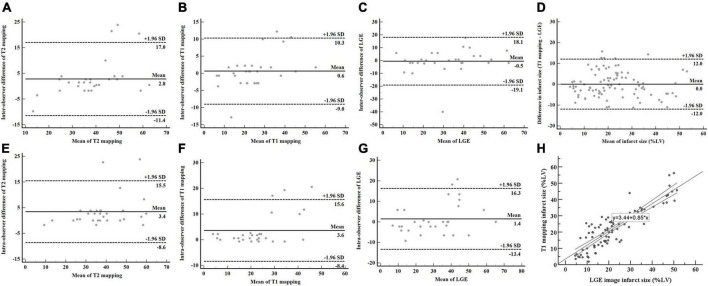
AAR and IS assessed by T2 mapping, T1 mapping, and LGE. The intraobserver and interobserver reproducibility of T2 mapping **(A,E)**, T1 mapping **(B,F)**, and LGE **(C,G)** were good. Bland-Altman analysis showed good agreement in measuring IS between T1 mapping and LGE **(D)**. T1 mapping correlated well with LGE in quantifying IS **(H)**. AAR, area at risk; IS, infarct size; LGE, late gadolinium enhancement.

Quantification of IS and AAR revealed good reproducibility. The intraobserver variability in measuring IS on T1 mapping and LGE were excellent, with ICCs of 0.956 (95% CI: 0.908–0.979), and 0.939 (95% CI: 0.872–0.971), respectively (Figures 6B,C). The interobserver variability in measuring IS on T1 mapping and LGE were good, with ICCs of 0.901 (95% CI: 0.727–0.958) and 0.907 (95% CI: 0.804–0.956), respectively (Figures 6F,G). The ICC for the intraobserver variability ranged between 0.925 (95% CI: 0.798–0.968) for T2 mapping (Figure 6A). The ICC for the interobserver variability ranged between 0.895 (95% CI: 0.770–0.951) for T2 mapping (Figure 6E). Bland-Altman analysis showed good agreement between T1 mapping and LGE for measuring IS (limits of agreement = 0.04 ± 6.13%) (Figure 6D). T1 mapping correlated well with LGE in quantifying IS (y = 3.44 + 0.85x, *R*^2^ = 0.765, *P* < 0.001) (Figure 6H).

## Discussion

Obstruction or relative insufficient supply of blood flow leads to myocardial necrosis and myocytolysis, transmembrane sodium gradients alteration, or inflammatory response after an acute ischemic insult leads to tissue edema and increased intersarcomeric distance (28, 29). The process of reperfusion therapy is a double-edged sword that accounts for salvaged myocardium; paradoxically, it is also a driving factor that contributes to additional myocardial damage (30). In patients with STEMI, myocardial AAR is the proportion of myocardium that is at risk of becoming necrotic, supplied by occluded artery, and it exceeds irreversibly infarct myocardium (13). The amount of salvaged myocardium is derived by subtracting IS from the edematous area (4). CMR is a promising tool that offers robust quantification of reversible and irreversible myocardial injury through analyzing T2w-STIR and LGE; thus, enabling us to determine the amount of salvaged myocardium, and it has been proven to show good consistency with SPECT and histological examinations (11, 31–34).

To the best of our knowledge, this is the first evaluation of MS using CMR mapping sequences. The major findings are as follows: (1) patients with < median MSI have larger IS, lower LVEF, and a significantly higher incidence of MACE at 1-year follow-up; (2) Combining MSI with LVEF obtained a stronger predictive value of MACE rate; and (3) Increased MSI was associated with shorter reperfusion time and decreased IS; on the contrary, MSI correlated positively with LVEF. Reperfusion time, IS, and LVEF have been proven to be associated with adverse patient outcomes (1, 24), and they showed fair correlations with MSI; as a corollary, CMR mapping-derived MSI might serve as a novel and strong predictor of clinical outcomes in patients with STEMI after reperfusion. In our study, a shorter reperfusion time was associated with higher MSI, which predicted a reduced risk of MACE. This confirmed current guidelines for patients with STEMI to accept reperfusion therapy as early as they can (35). LVEF is a typically reliable marker that reflects cardiac function and clinical outcome, combining analysis with MSI increased the prognostic accuracy on the risk of MACE than LVEF itself.

More and more studies demonstrated native T1 mapping-enabled estimation of IS in animal models and patients (15, 36, 37). Messroghli et al. demonstrated that high-resolution T1 mapping enabled the detection of AMI; standard T1 relaxation time thresholds served as a potential tool for the measurement of IS with high sensitivity and specificity (15). Cui et al. proved that the presence and extent of myocardial infarction measured by T1 mapping correlated well with that evaluated by LGE and triphenyl tetrazolium chloride staining in the porcine study (14). Not only in AMI but T1 mapping was also validated as amenable in chronic myocardial infarction (38). Thus, native T1 mapping constitutes a surrogate method for contrast agent-dependent technique. This advantage broadens CMR application by reducing scanning time and especially benefits patients with renal dysfunction.

T2 is sensitive to myocardial water content, edema in infarct tissue prolongs T2 signal decay and depicts a hyperintense region. Aletras et al. showed that increased signal area measuring T2-weighted imaging was comparable with the AAR detected with microspheres in coronary occluded animal models (39). Ugander et al. confirmed that the histologic AAR and T2-defined AAR overlapped to a large extent in the canine infarction model (40). T2 mapping provides estimated T2 values per pixel in milliseconds, and it has been validated to serve as an attractive approach to measuring myocardial edema (33). Traditional T2 sequences introduce several limitations, on the one hand, mistiming the image scanning outside the diastolic R-R interval leads to signal reduction, while on the other hand, remaining blood caused by trabeculae in the ventricular cavity obscures the borders of the myocardium and increases signal intensity. T2 mapping overcomes the aforementioned limitations (41).

### Limitations

T2 values change dynamically over the first few days after STEMI. Bimodal edema pattern has been illustrated in human studies (34). The first peak develops directly after reperfusion; it is caused by cell swelling and reactive hyperemia with capillary leakage, whereas the second peak occurs from 4 to 7 days after PPCI, and results from tissue inflammation and regeneration. Thus, T2 mapping is still under debate for robust evaluation of AAR. Native T1 mapping has been proven to underestimate IS by 10% as validated by histological examination in swine experiments (14). However, histologic findings in animal models cannot directly represent the conditions in the human body. Moreover, microvascular damage, ranging from microvascular obstruction to intramyocardial hemorrhage, always overlaps with edema, thus mitigating T1 prolongations, and leading to underestimation of the resultant T1 value (13). Further clinical trials and multiple parallel compare methods are needed to verify the potential of native T1 mapping in quantifying irreversible infarct areas. This study was conducted in a single center with a small cohort. In addition, only three short-axis slices (basal, mid, and apical) were scanned and analyzed in T1 and T2 mapping, we suppose that more slices covering from ventricular base to apex might increase accuracy in measuring salvaged myocardium.

## Conclusion

This work emphasizes the promising prognostic role of contrast agent-free CMR sequences in providing *in vivo* characterization of myocardial tissue damage in patients with STEMI. MSI contributes to assessing the efficacy of reperfusion therapy and increases the predictive value of the MACE rate in reperfused myocardial infarction.

## Data availability statement

The raw data supporting the conclusions of this article will be made available by the authors, without undue reservation.

## Ethics statement

The studies involving human participants were reviewed and approved by the Committee of The Second Affiliated Hospital of Harbin Medical University (Harbin, China). The patients/participants provided their written informed consent to participate in this study.

## Author contributions

YLL and SL designed the study and wrote the manuscript. XYW and YL post-processed the images. XG, JC, and BX collected the data. XDW, GW, and YZ analyzed the data. YS and BY supervised the study. All authors read and approved the final manuscript.
